# Conditions of Participation: Incorporating the History of Hospital Desegregation

**DOI:** 10.1017/jme.2024.13

**Published:** 2023

**Authors:** Sallie Thieme Sanford

**Affiliations:** 1. UNIVERSITY OF WASHINGTON SCHOOL OF LAW, SEATTLE, WASHINGTON, USA.

**Keywords:** Simkins, Hospital Desegregation, Civil Rights Act, Medicare, Civil Rights History

## Abstract

Our students ought to know about the history of formal hospital segregation and desegregation. To that end, this article urges those who teach foundational health law and policy courses to do three things. First, to teach the Simkins case. Second, to swap out the usual Medicare signing ceremony picture for one that includes W. Montague Cobb, M.D., Ph.D. Third, to highlight how the implementation of that program for the elderly led, in a matter of months, to the desegregation of hospitals throughout the country.

When I was a newly minted, practicing healthcare attorney, my father related a story from his time as a medical student in St. Louis, Missouri. It happened, he said “at the black hospital.” What, I asked, was a black hospital, hoping it had to do with the building’s cladding. “The hospital for Black people. Don’t they teach you anything at those schools you’ve been to?”

At the many schools I attended, I had learned, of course, something about our country’s history of *de jure* and *de facto* racial segregation but, no, I had not been taught that history as applied to hospitals. I did not know about the prominent role of Black hospitals, about the Hill–Burton Act’s financial support of racially segregated facilities, or about the *Simkins v. Moses H. Cone Memorial Hospital* decision.

I did not know about the *Simkins*-grounded arguments that advanced passage of Title VI of the Civil Rights Act of 1964. I did not know about the healthcare justice movement’s crucial advocacy for Medicare and Medicaid. And I hadn’t heard the story of how bureaucrats, activists, and policymakers leveraged the initial Medicare certification process to rapidly end formal racial segregation in hospitals.

Decades later, most of my law school and Masters of Health Administration (MHA) students arrive at their foundational health law class similarly uniformed. Racial nondiscrimination is a condition of participation in Medicare. Learning about this history ought to be a condition of participation in introductory health law and policy courses.

To that end, this article urges those who teach these courses to do three things. First, to teach the *Simkins* case. Second, to swap out the usual Medicare signing ceremony picture for one that includes W. Montague Cobb, M.D., Ph.D. Third, to highlight how the implementation of that program for the elderly led, in a matter of months, to the desegregation of hospitals throughout the country.

## Advancing Pedological Goals

Teaching this history early on in a foundational class advances important pedological goals. First it highlights, in a readily graspable way, the law as a factor in healthcare disparities, an example of a political determinant of health, for good and for bad. Relatedly, it elevates the place of impacted communities in promoting legal change in the healthcare arena. Second, it reinforces the point that law comes from a variety of sources. The synergistic legal tools utilized here include statutes, regulations, judicial opinions, litigation strategy, implementation decisions, and private responses.

Third, it provides an example of change supported and opposed by those in professional roles to which our students aspire. Lawyers, administrators, regulators, and organizers feature prominantly. Fourth, it advances student understanding of staple substantive topics: hospital evolution, privileging, the Hill–Burton Act, Medicare and Medicaid, anti-discrimination law, and congressional spending powers. In my experience, it need not require much extra time or curricular revision to deepen these topics with a civil rights era context.

Finally, incorporating this history aligns with current accreditation standards. Law schools, for example, must educate their students about “bias, cross-cultural competency, and racism,” and provide substantial opportunities for “the development of a professional identity,” including the obligation to work towards the elimination of bias, racism, and discrimination in the law.^1^

## Teach *Simkins v. Moses H. Cone Memorial Hospital*

The mid-1960’s were an inflection point in the ongoing quest for healthcare justice. An instructive aspect of that quest is the *Simkins* case. George Simkins, Jr., D.D.S. practiced dentistry in Greensboro, North Carolina and served for many years as head of the local chapter of the National Association for the Advancement of Colored People (NAACP).

In 1962 Dr. Simkins, joined by several physicians and patients and represented by NAACP lawyers, sued two private, not-for-profit hospitals in Greensboro, arguing that their segregationist practices violated the Constitution. Both defendant hospitals had received federal Hill–Burton funds pursuant to North Carolina’s hospital expansion plan that specified racially segregated facilities. So, too, had the city’s other hospital, L. Richardson Memorial Hospital, the smallest of the three, which served Black patients and where Black physicians, including the plaintiffs, held privileges.

The federal district court dismissed the case on summary judgment, holding that the Constitution’s equal protection requirements did not apply to these private entities. The plaintiffs’ appeal was highly unusual in at least two respects. First, the appellate court decided on its own initiative to hear the case *en banc.* Second, the U.S. government intervened in support of the plaintiffs.

In November 1963, the Fourth Circuit handed down a 3–2 decision holding that the hospitals’ involvement with the Hill–Burton program provided the requisite “state action” to bring them within the Constitution’s equal protection requirements.^2^ Thus, they were required to desegregate: to admit patients, employ people, and privilege physicians without regard to race. The court also ruled unconstitutional the Hill–Burton Act’s “separate but equal” provision, which had been a dramatic example of federal support for Jim Crow regimes.

The hospitals petitioned the U.S. Supreme Court to take the case. The American Medical Association (AMA) and the American Hospital Association (AHA) filed *amicus* briefs in support of the hospitals. The Supreme Court in early March 1964 denied *cert*.^3^ Thus, future recipients of Hill–Burton funds could not racially discriminate. And *Simkins*-style lawsuits could be brought to force desegregation of private hospitals that had received this federal funding in the past.

At the same time, across the street, Congress was debating what would become the Civil Rights Act of 1964 (CRA), including its important provision (Title VI) barring racial and other discrimination by any recipient of federal funds. In successfully arguing for its passage, advocates highlighted the *Simkins* holding and the Supreme Court’s decision to let it stand.^4^

## *Simkins* Case Materials

Early on in my foundational law and MHA classes, I assign an edited version of the Fourth Circuit’s majority opinion;^5^ the full opinion, including dissent, is not long and could be assigned in whole. Depending on the students’ background knowledge, I might tee it up with historical and procedural background set out on PowerPoint slides. I provide reflective prompts and have the students either post a written response or prepare to discuss in class. These teaching resources and others discussed in this essay are available as an on-line appendix.^6^

I also assign the 2019 documentary film, *The Power to Heal: Medicare and the Civil Rights Revolution*,^7^ which includes discussion of the case. Other useful internet resources (including a short documentary) center around the 2016 commemoration of the case by Cone Health Network and could spark interesting discussions about the uses and substance of historical markers, monuments, and formal apologies.

This year I incorporated artificial intelligence (A.I.) analysis. I tasked ChatGPT and two other popular A.I. programs with briefly summarizing and contextualizing *Simkins* and offered students the opportunity to critique the responses. All the A.I. responses seemed logical, but there were stupendous errors: one stated that the court’s holding was grounded in federal tax exemption requirements; another described the plaintiff as nursing student Elizabeth Simkins suing to enforce the CRA. Of course, *Simkins* predates the CRA, whose Title VI undergirded the groundbreaking implementation efforts of another piece of Great Society legislation.

## Swap out the Usual Medicare Signing Photo

All introductory health law and health policy courses discuss the 1965 enactment of Medicare and Medicaid. And they often illustrate this milestone with a photo of former President Harry S Truman at President Lyndon B. Johnson’s side during the signing ceremony. This ubiquitous photo is a good one, and it nicely illustrates the multi-administration, politically challenging effort towards universal coverage.

I urge the use of a different photo. This different one is from the same event, taken from the same general vantage, and still includes both presidents. Its focus, though, is on President Johnson talking to Dr. Cobb. Dr. Cobb, a noted advocate for healthcare non-discrimination, served for many years as the head of the National Medical Association (NMA). The NMA was founded in 1895 as the voice of Black physicians; the organization was represented at the signing ceremony by Dr. Cobb, who was specifically invited. Notably absent from this landmark celebration were any members of other physician or hospital or insurance organizations.

This replacement picture helps illustrate the key role of the civil rights movement in the quest for universal healthcare access. Dr. Cobb was the sole leader of a medical association to testify in favor of Medicare. (The AMA and AHA vigorously opposed it.) Civil rights leaders and activists were instrumental in the legislation’s passage, with both public actions and private lobbying. Indeed, there seems something of a *quid pro quo* with the NMA’s support for Medicare and the administration’s support for ending legalized hospital segregation.

The replacement photograph is not as nicely composed as the standard one, but that fact hints at some of the political maneuvering and behind-the-scenes work. Despite their importance to the legislative victory, neither Dr. Cobb nor the NMA were recognized in the formal remarks or in the staged photos. If President Truman was, in President Johnson’s words, “the real daddy of Medicare,”^8^ civil rights advocates, as represented by Dr. Cobb, served as its midwife—a midwife who was kept in the background, perhaps for strategic, political reasons.

## Photo with Dr. Cobb

With thanks to the reference librarians at the LBJ Presidential Library for uncovering it, the photo with Dr. Cobb is reproduced below and is also included as part of an available PowerPoint in the online appendix at endnote 6 below.

**Figure 1:**
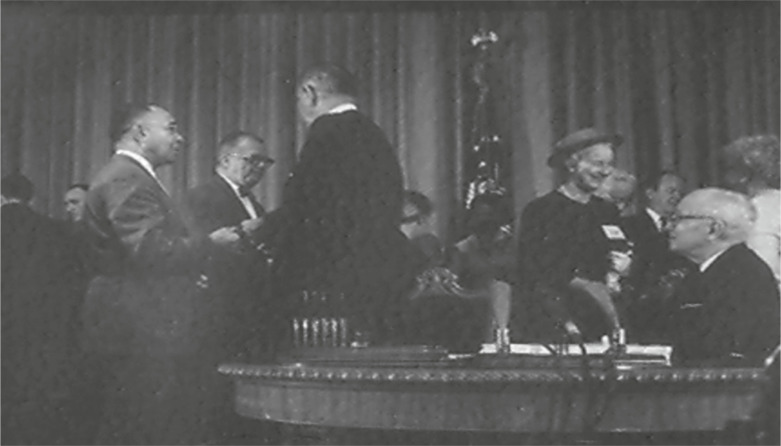
W. Montague Cobb, M.D., Ph.D., representing the National Medical Association, with President Lyndon B. Johnson and former President Harry S. Truman (seated) at the signing ceremony for the Medicare and Medicaid legislation. White House photo by Yoichi Okamoto, courtesy of LBJ Presidential Library Archives.

## Highlight Medicare’s Role in Hospital Desegregation

The *Simkins* case offers lessons in the power of litigation, and the Great Society statutes highlight the force of legislation. Less familiar to most students is another legal tool with transformative protentional: agency implementation. This regulatory function shines in the use of Medicare’s initial certification process to drive hospital desegregation.

That use was not a foregone conclusion. Medicare was set to go live in July 1966; thus, within a year of the signing ceremony, the department of Health, Education and Welfare (HEW, predecessor to the Department of Health and Human Services) needed to enroll not only millions of beneficiaries, but also tens of thousands of physicians, and thousands of hospitals. In the face of continuing opposition from the AMA and AHA and the ubiquity of segregated healthcare, a possible strategy was to sideline racial justice in the press to roll out this marque program.

Indeed, for the first many months, that incrementalist approach seemed a distinct possibility, prompting Black medical leaders and the NAACP to publicly blast HEW. One result of their activism was the January 1966 establishment of the HEW Office of Civil Rights (OCR), staffed by many people with direct experience in that movement. This added internal pressure for an administrative strategy that conditioned Medicare payments on evidence of desegregation, at least as to hospitals.

In late Spring, 1966, OCR adopted an innovative approach that engaged hundreds of volunteers from throughout HEW to form teams of hospital inspectors. After training that included agency lawyers and civil rights activists, these teams made site visits to document conditions and explain the program’s requirements. In some areas, they worked with community members and hospital workers to expose the “HEW shuffle” of segregationist signs temporarily hidden under pictures and sham patients posed to suggest integrated wards. In addition to subterfuge, in some locales these inspectors faced physical risks including intimidation and death threats.

With a month to go, most northern hospitals, but fewer than half of those in the south, were on board. In some regions, including large metro areas, the leading hospitals maintained their practice of denying, limiting, or segregating Black patients, employees, and medical staff. HEW readied backup plans to transport Medicare patients to federal facilities. And President Johnson summoned hospital leaders to the White House and told them that his administration was firm on this: if they wanted Medicare money, they would have to comply with the civil rights law.

By the launch date, July 1, 1966, more than 90 percent of the nation’s hospitals and over 70 percent of those in the South were deemed in compliance.^9^ Formal racial segregation in hospitals had been nearly eliminated in a matter of months. Working synergistically, bureaucrats, community members, activists and policy leaders had scored a huge, albeit still incomplete, victory for healthcare justice.

## Hospital Desegregation Resources

An excellent way to teach this history and its context is to assign the previously referenced 2019 documentary film, *The Power to Heal: Medicare and the Civil Rights Revolution*, perhaps with pre-class reflective prompts. The film is available (in electronic and DVD formats) from many university libraries and might soon be rebroadcast on PBS. At about an hour long, the film could be shown during class. If in-class and pre-class time is scarce, even showing the two-and-a-half-minute trailer could advance student knowledge.

The film’s website includes, among other resources, stand-alone videotaped interviews. The book that served as an inspiration for the documentary includes an extensive bibliography which could provide resources for a seminar or focused course module.^10^ And this history offers a springboard to the burgeoning literature about health disparities and ways to ameliorate them.^11^ It also invites discussion of the development of Black hospitals, ^12^ and how desegregation soon led to the closure of most. Those closures and the emergence of more subtle forms of racial discrimination in access to medical care and insurance coverage had repercussions for Black healthcare professionals, patients, and many community-based healthcare facilities.^13^

## Conclusion

The Black facility in St. Louis that my father referenced when he bemoaned my lack of knowledge was the Homer G. Phillips Hospital. Built in the 1930’s after a campaign by a prominent Black attorney (for whom it was later named) and other community leaders, it became one of the largest general hospitals in the country. In addition to serving Black patients, it was for decades a leading training site for Black physicians, nurses, and other healthcare professionals, a source of community pride and comfort.

It was also a casualty of *de jure* integration and other changes, as it was substantially absorbed into better-resourced hospitals that had predominantly served white patients and primarily privileged white physicians. Despite community protests, it closed completely in the 1970s.^14^ Had I been taught the history of hospital segregation and desegregation, I could have intuited much of this.

The *Simkins* decision is not widely known. On its face, the Civil Rights Act is not healthcare legislation. Medicare is not, *per se*, a civil rights law. And the 1966 hospital inspection work is not heralded in the era’s historiography. All, though, were crucial factors in the ongoing, complex quest for healthcare justice. As a condition of participation in foundational health law and policy courses, our students should learn about this history.

## Supplementary material

For supplementary material accompanying this paper visit https://doi.org/10.1017/jme.2024.13. click here to view supplementary material
