# Supplementary material for: Conditions of Participation: Incorporating the History of Hospital Desegregation
Source: J Law Med Ethics. 2023 Winter;51(4):979–83. doi: 10.1017/jme.2024.13 (PMC10937164; doi:10.1017/jme.2024.13)
Supplement: Supplementary file 1 [file S1073110524000135sup001.pdf]

Sallie Thieme Sanford

Conditions of Participation:
Incorporating the History of Hospital Desegregation
OnLine Appendix

Included here:

- Edited *Simkins* Decision
- *Simkins* Reflective Prompts
- *Power to Heal* Reflective Prompts
- PowerPoints including *Simkins* Legal Background and Medicare Signing Photos

Edited *Simkins* decision
This is an edited version of the landmark desegregation case, *Simkins v. Moses H. Cone Memorial Hospital*, 323 F.2d 959 (4th Cir. 1963) (*en banc*), *cert denied*, 376 U.S. 938 (1964). This edited version is published in J.S. Showalter and S.T. Sanford, *The Law of Healthcare Administration* at 28 – 30 (tenth edition, AUPHA/Health Administration Press, 2023). Note that internal citations have been omitted.

Simkins v. Moses H. Cone Memorial Hospital
323 F.2d 959 (4th Cir. 1963) (en banc)

Sobeloff, Chief Judge

The threshold question in this appeal is whether the activities of the two defendants, Moses H. Cone Memorial Hospital and Wesley Long Community Hospital, of Greensboro, North Carolina, which participated in the Hill-Burton program, are sufficiently imbued with "state action" to bring them within the Fifth and Fourteenth Amendment prohibitions against racial discrimination. Beyond this initial inquiry lies the question of the constitutionality of a portion of the Hill-Burton Act. . . . Because of the importance of these questions the court, on its own motion, has heard the appeal en banc. *[This means that the appeal was considered by all the judges on the Fourth Circuit, not the usual three-judge panel.]*

The plaintiffs are Negro physicians, dentists and patients suing on behalf of themselves and other Negro citizens similarly situated. . . . The basis of their complaint is that the defendants have discriminated, and continue to discriminate, against them because of their race in violation of the Fifth and Fourteenth Amendments to the United States Constitution. The plaintiffs seek an injunction restraining the defendants from continuing to deny Negro physicians and dentists the use of staff facilities on the ground of race; an injunction restraining the defendants from continuing to deny and abridge admission of patients on the basis of race, . . . and a judgment declaring unconstitutional [the portions of the Hill-Burton Act and its implementing regulations] which authorize the construction of hospital facilities . . . on a 'separate-but-equal' basis. . . .

[Because the complaint challenges the constitutionality of a federal statute and affects the public interest, the United States moved to intervene (requested to participate) in the

proceeding.] Its motion for intervention was granted and throughout the proceedings the [Federal] Government, unusually enough, has joined the plaintiffs. . . .

. . . .

Factual Background

. . . .

The claims of racial discrimination were, as the District Court found, "clearly established." In fact the hospitals' applications for federal grants for construction projects openly stated, as was permitted by [the Hill-Burton] statute, and regulation, . . . that 'certain persons in the area will be denied admission to the proposed facilities as patients because of race, creed or color.' These applications were approved by the North Carolina Medical Care Commission, a state agency, and the Surgeon General of the United States under his statutory authorization.

. . . .

When this action was commenced, the United States had appropriated $1,269,950.00 to the Cone Hospital and $1,948,800.00 to the Long Hospital. . . . These appropriations for the most part were after the Supreme Court's landmark decisions in Brown v. Board of Education *[the 1954 and 1955 US Supreme Court decision that overruled its prior precedent and unanimously held that the Fourteenth Amendment prohibits states from segregating students by race]* . . . .

. . . .

The point of present interest is not the equality or lack of equality in "separate-but-equal," but the degree of participation by the national and state governments. *[Though, in a footnote, the court cited a 1962 report finding that in North Carolina the hospitals "available to nonwhites were both inferior to those available to whites and more limited."]*

The Legal Issue

In our view the initial question is . . . whether the state or the federal government, or both, have become so involved in the conduct of these otherwise private bodies that their activities are also the activities of these governments and performed under their aegis without the private body necessarily becoming either their instrumentality or their agent in a strict sense. . . .

[The court recognizes that purely private action would not violate the Constitution, but concludes that there is state action here, pointing to the "massive use of public funds and extensive state-federal sharing in the common plan" inherent in the Hill-Burton program's functioning. The test for what constitutes "state action" is different now; this is discussed further in chapter 14.]

. . . .

Moreover, the [Federal] Government's argument stresses the fact that the challenged discrimination has been affirmatively sanctioned by both the state and the federal government pursuant to federal law and regulation. . . . These federal provisions undertaking to authorize segregation by state-connected institutions are unconstitutional. . . . Unconstitutional as well under the Due Process Clause of the Fifth Amendment and the Equal Protection Clause of the Fourteenth are the relevant regulations implementing this passage in the statute.

[The court discusses the hospitals' counterarguments and dismisses them.] Not only does the Constitution stand in the way of the [defendant hospitals'] claimed immunity but there are powerful countervailing equities in favor of the plaintiffs. Racial discrimination by hospitals visits severe consequences upon Negro physicians and their patients. *[In a footnote, the court supports this statement by noting that "[r]acial discrimination in medical facilities is at least partly responsible for the fact that in North Carolina the rate of Negro infant mortality is twice*

the rate for whites and maternal deaths are five times greater." Furthermore "[e]xclusion of Negro physicians from practice in hospitals on account of their race denies them opportunities for professional improvement and has discouraged Negro physicians from practicing in the cities of the South."]

Giving recognition to its responsibilities for public health, the state elected not to build publicly owned hospitals, which concededly could not have avoided a legal requirement against discrimination. Instead it adopted and the defendants participated in a plan for meeting those responsibilities by permitting its share of Hill-Burton funds to go to existing private institutions. The appropriation of such funds to the Cone and Long Hospitals effectively limits Hill-Burton funds available in the future to create nonsegregated facilities in the Greensboro area. In these circumstances, the plaintiffs can have no effective remedy unless the constitutional discrimination complained of is forbidden.

The order of the District Court is reversed. *[Two judges joined the chief judge's opinion, forming a narrow majority; two judges dissented, "[b]believing the majority both unprecedented and unwarranted."]*

Simkins Reflective Prompts

1. Why has this case been called "the *Brown v. Board of Education* decision for hospitals"? How is it like *Brown*? How is it different?
2. This was the first case in which the federal government intervened to argue that a federal statute was unconstitutional. Noting particularly the date of this case, consider what factors might have influenced the US Department of Justice to intervene in this case, advocating for the plaintiffs' position.
3. The dissent noted that in August 1963, just a couple of months before this decision was handed down, "the Senate rejected a proposal that henceforth grants in aid to hospitals under the Hill-Burton Act be restricted to hospitals which are desegregated, and which practice no discrimination on account of race." Does that surprise you?
4. The US Supreme Court "denied cert" in this case. What does that mean? What, in general, is the practical consequence of that action? What, in particular, do you know or suspect were the consequences of this cert denial?
5. With informal reference to the edited *Simkins* decision, critique one of the AI responses.

***Power to Heal* Reflective Prompts**

1. What legal tools were utilized in this desegregation effort?
2. What role did hospital administrators, hospital board members, and medical associations play?
3. How does the film mesh or not with your prior knowledge?
4. Is there a phrase or image or person from the documentary that sticks with you?
5. Does the early Medicare certification process hold any lessons for current transformational efforts?
6. What other reflections about the documentary would you like to share?

PowerPoints including *Simkins* Legal Background and Medicare Signing Photos

Conditions of Participation Appendix PowerPoints

Sallie Thieme Sanford
Associate Professor
University of Washington School of Law

A Turning Point in the Quest for Healthcare Justice

- Trailer to the video (note how to watch)
- Linked to Simkins case

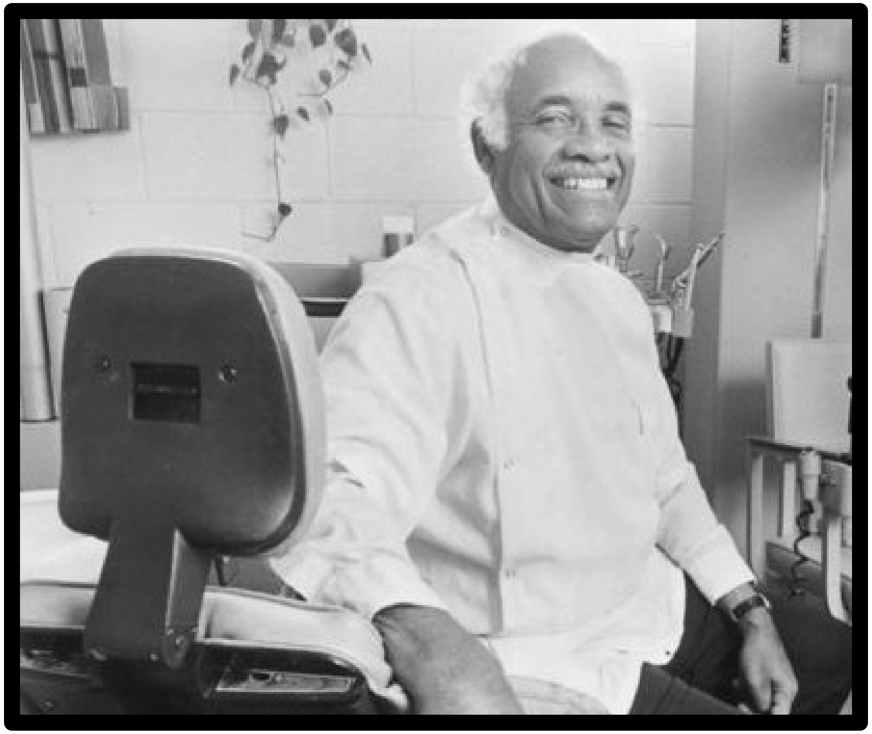

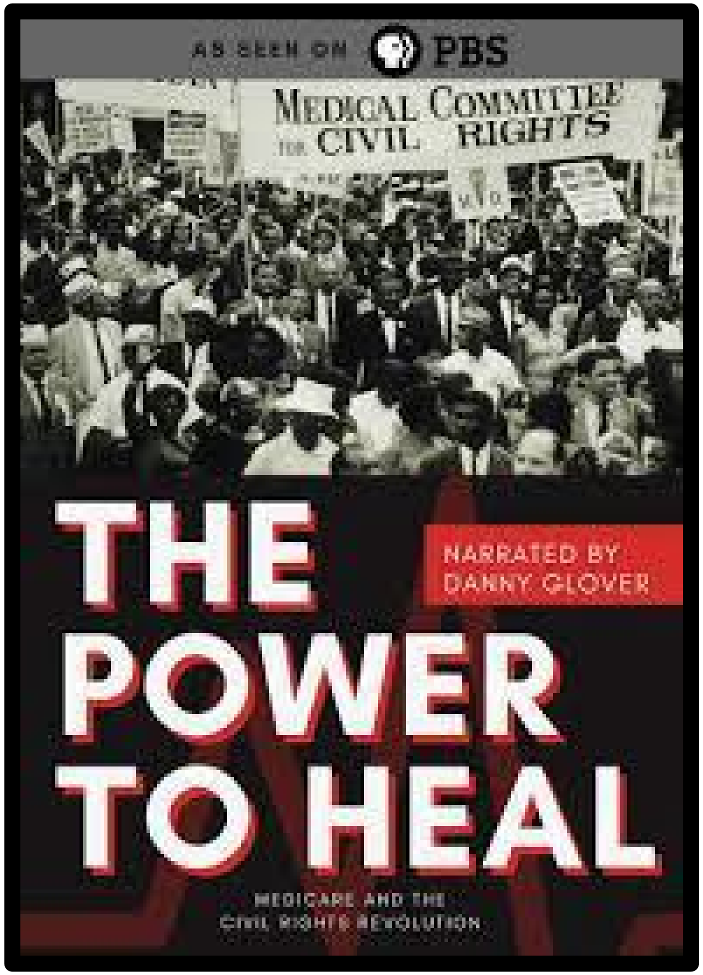

Law Timeline Discussed in Video

- 1868 14th Amendment ratified
- Jim Crow laws in various states
- Racial segregationist private actions
- 1896 Plessy v. Ferguson
- 1946 Hill-Burton Act, and Regulations
- 1954, 1955 Brown v. Board of Education
- 1963, 1964 Simkins v. Cone Hospital
- 1964 Civil Rights Act enacted, including Title VI
- 1965 Medicare enacted
- July 1966 Medicare goes live

Some of this is probably familiar to you from prior studies.

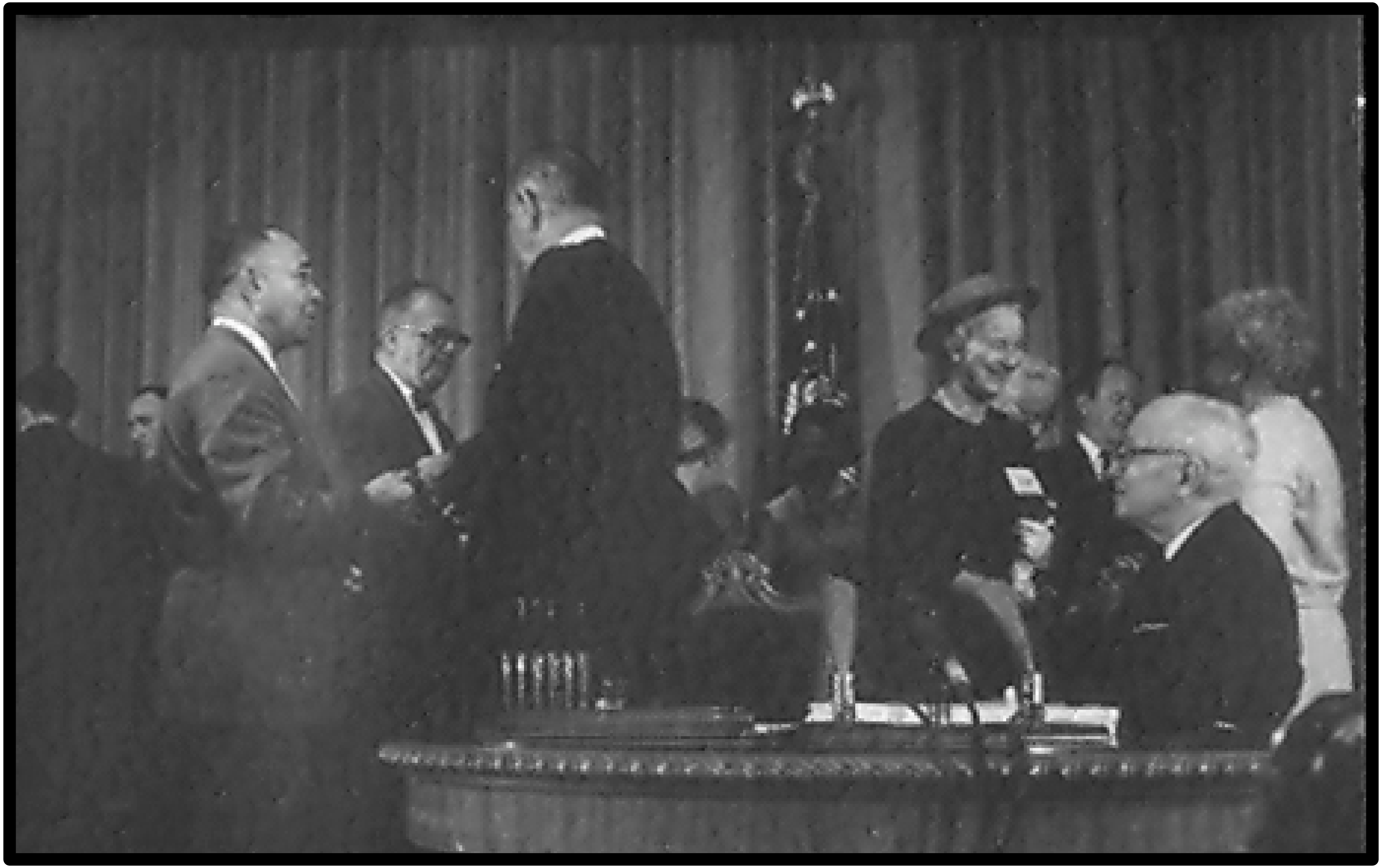

W. Montague Cobb, MD, PhD, head of the National Medical Association, with President Lyndon B. Johnson and former President Harry S Truman (seated) at the signing ceremony for the Medicare and Medicaid legislation. LBJ Presidential Library Archives

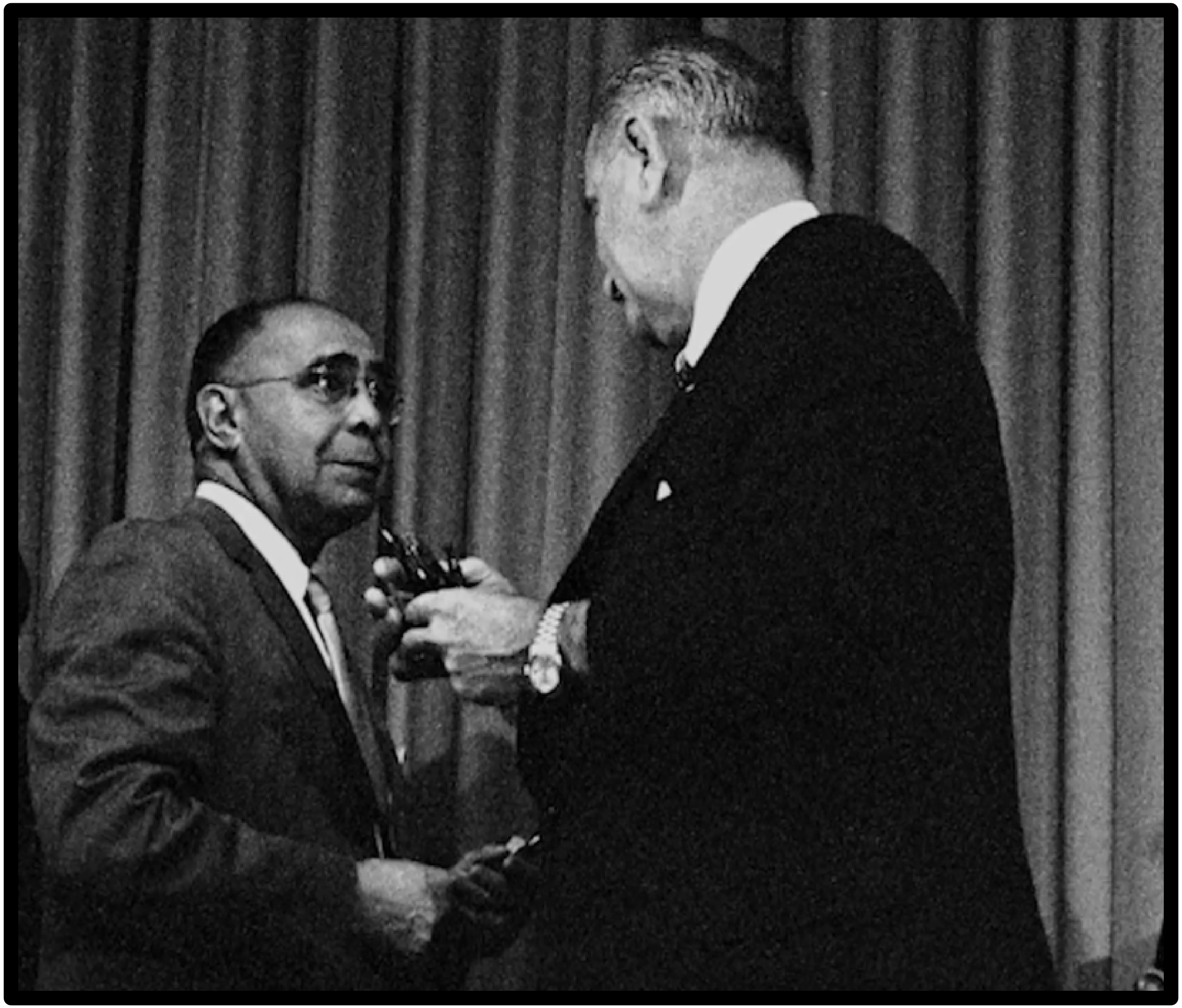

W. Montague Cobb, MD, PhD, head of the National Medical Association, with President Lyndon B. Johnson at the signing ceremony for the Medicare and Medicaid legislation. *The Power to Heal: Medicare and the Civil Rights Revolution* (Bullfrog Films 2019) at 38:41. LBJ Presidential Library Archives.
